# Genetics and orofacial clefts: a clinical perspective

**DOI:** 10.1038/s41415-023-5994-3

**Published:** 2023-06-23

**Authors:** Usha Kini

**Affiliations:** Oxford Centre for Genomic Medicine, Oxford University Hospitals, UK; Spires Cleft Service, Oxford University Hospitals, UK; NDCLS, Radcliffe Department of Medicine, University of Oxford, United Kingdom

## Abstract

Orofacial clefts (OFCs) are the most common congenital craniofacial anomaly seen in humans. Most OFCs are sporadic and isolated - these are thought to be multifactorial in origin. Chromosomal and monogenic variants account for the syndromic forms and for some of the non-syndromic inherited forms. This review discusses the importance of genetic testing and the current clinical strategy to deliver a genomics service that is of direct benefit to patients and their families.

## Key points

- Genetics and genomics are the most rapidly advancing fields of medicine. This paper discusses these advances in the context of clefts.
- The paper gives a clear classification of clefts based on aetiology.
- Clinicians from non-genetic specialities develop an understanding of the utility of genetics in the management of their patients and families.

## Introduction

Orofacial cleft (OFC) is the most common craniofacial malformation in humans. The incidence varies geographically and anatomically by the cleft subtype. For example, the incidence of cleft lip and palate (CLP) is reported to be 1 in 700 and that of cleft palate only (CPO) is 10-25 in 100,000. Embryologically, CLP and CPO occur consequent to a disruption to separate processes, which likely explains the difference in the incidence of these cleft subtypes. The geographic variation in the incidence is thought to be multifactorial, particularly related to lifestyle factors, such as alcohol intake, smoking and folate deficiency, and inherent genetic susceptibility factors. Overall, a higher incidence has been reported in Japan and Bolivia.^1^

Despite its frequency, the aetiology of clefting is not fully understood. Several environmental factors have been implicated and genetic associations for both non-syndromic and syndromic clefting have been suggested, but a comprehensive understanding has not yet been established. The importance of understanding the aetiology of OFCs is to firstly answer a parent's or affected individual's question of 'why and how did this happen?' and secondly, to consider appropriate intervention and prevention measures, primarily for the affected family and then more widely for the general population.

The majority of OFCs are isolated (or non-syndromic), with only 30% of all clefts being thought to be syndromic.^2^^,^^3^ Genetic forms of non-syndromic CLPs are far more common than those of CPO. On the other hand, CPO is more often seen as a syndromic feature compared to CLP. However, with improved accessibility to advanced genomics technology, the list of monogenic cause of syndromic CLP is also growing.

## Embryology

Embryologically, the development of the upper lip and palate is complete by the first trimester. Failure of the normal processes of migration of cells, and growth and fusion of adjacent facial tissues, results in a cleft. At approximately seven weeks of gestation, the maxillary processes fuse with the medial nasal eminences forming the labial grooves.^4^ Migration of mesenchymal cells to fill these grooves results in normal lip morphology. A disruption to either or both these processes results in a cleft lip.

Normal palate morphology is dependent on the fusion of the mesenchymal lateral palatine processes with each other, the nasal septum, and the posterior margins of the median palatine processes. Anomalous fusion of any of these structures can result in a cleft palate.

Pierre Robin sequence (PRS) refers to the triad of wide U-shaped cleft palate, micrognathia and glossoptosis (retro-positioning of the tongue). It occurs as a sequence of events that stem from the formation of a small chin (micrognathia). During normal development, the tongue descends into the oral cavity, allowing room for the fusion of palatal processes with the nasal septum. With micrognathia, the oral cavity remains small, with insufficient room for the normal-sized tongue to descend. This obstructs the fusion of palatal processes, resulting in the wide cleft palate. The normal-sized tongue, which is large for the small oral cavity, then falls backwards and has the potential to compromise the patient's airways.

## Aetiology

The anatomical classification of the cleft is important while considering its genetic causes. For example, a midline cleft (Tessier number 0) may indicate a holoprosencephaly spectrum, genes that affect midline development (for example, MID1), or specific syndromes, such as oro-facial-digital syndrome or Ellis-van Creveld syndrome. A lateral cleft resulting in macrostomia on the other hand may imply genes related to Treacher Collins syndrome (TCOF1, POLR1C, POLR1B, POLR1D).^5^ The spectrum of a cleft lip may stretch from a small notch in the upper lip (*forme fruste*) to unilateral cleft lip, unilateral CLP, and extensive bilateral involvement affecting the nostrils, gums and palate. Cleft palate, on the other hand, may stretch from a bifid uvula to a submucous cleft palate, cleft soft palate, or a wide complete cleft palate.^6^ Hence, careful history-taking and examination is important when considering a familial or syndromic cleft.

PRS may be isolated or may be syndromic. The non-syndromic forms of PRS may have an environmental aetiology, such as oligohydramnios. The three main genetic causes of syndromic PRS are SOX9 variants, chromosome 22q11 deletion and Stickler syndrome.^7^ Stickler syndrome is commonly caused by variants in the COL2A1 or COL11A1 genes, where ophthalmic complications, such as retinal detachment and blindness, may be seen; the retinal detachment can be prevented by retinopexy in those with a fragile retina. An early diagnosis is therefore crucial in enabling management of symptoms that affect the quality of life of these patients. Many other genes known to affect the development and growth of the mandible resulting in micrognathia are associated with PRS, for example, SF3B4 gene causing Nager syndrome and SNRPB gene causing cerebrocostomandibular syndrome.

Isolated or non-syndromic clefting (more commonly CLP) is usually sporadic in nature but may sometimes have a monogenic cause. Sporadic OFCs show multifactorial aetiology which implies an interaction between genetic susceptibility factors and environmental factors, for example, alcohol exposure, antiepileptic drug exposure,^8^ folate deficiency and genetic susceptibility factors. Genome-wide association studies^9^^,^^10^ and epigenome-wide association studies (EWAS)^11^have been carried out to understand these genetic susceptibility factors better. Over 40 loci (single nucleotide polymorphisms)^12^ have been identified in association with non-syndromic clefting and the EWAS studies have identified distinct methylation signatures between the cleft subtypes.^13^ Although these studies enhance our understanding of the aetiology and formation of OFCs, at the present time, this information cannot be used clinically in the management of patients born with a cleft.

Genetic forms of non-syndromic OFCs usually show an autosomal dominant pattern of inheritance and may show reduced penetrance and variable expressivity. The most common monogenic cause of non-syndromic CLP (NSCLP) is variants in the IRF6 gene. This gene also causes Van der Woude syndrome, where lip pits, cysts and oral synechiae may be present, or the allelic popliteal pterygium syndrome, which is very rare (1 in 300,000) but much more severe in its presentation. There is no genotype-phenotype correlation recognised with IRF6 variants, the clinical implication of which is that an IRF6 variant identified on prenatal testing is unlikely to be able to predict the outcome in the baby. Variants in the GRHL3 gene are a rarer cause of NSCLP and can also cause Van der Woude syndrome 2 (VWS2) Examples of other genes that cause both syndromic and non-syndromic CLP include CDH1, GDF11, CTNND1A, TP63, TBX1, LRP6.^14^^,^^15^^,^^16^^,^^17^ Another known cause of NSCLP is the MSX1 gene, which may be associated with tooth agenesis.

Monogenic causes of non-syndromic cleft palate are rare. A recent study by Lace *et al.* showed that pathogenic and likely pathogenic variants in TBX22, COL2A1, FBN1, PCGF2 and KMT2D were identified in five patients in a small cohort of 30 patients with isolated cleft palate by whole genome sequencing.^18^ As these genes are well-known syndromic genes, this study reflects the variable expressivity seen with genes causing OFCs.

Syndromic clefting refers to OFCs that are associated with additional health problems, including other congenital malformations, growth problems, dysmorphic facies, developmental delay, intellectual disability and behavioural problems (for example, autistic spectrum disorder, attention deficit hyperactive disorder etc). Syndromic clefts may have a chromosomal cause, such as a deletion or duplication or other copy number variants of a single or multiple chromosomes; the latter is fortunately rare. A distinction between chromosomal aberrations causing syndromic CLP and syndromic CPO is observed, for example, some recurrent microdeletion/duplication syndromes, such as chromosome 22q11.2 deletion (Di George syndrome or velocardiofacial syndrome); chromosome 16p11.2 deletion syndrome is usually associated with CPO. The genes TBX1 and MAPK3 lying within these deletions, respectively, are thought to be causal in the cleft.^19^There are no specific recurrent chromosomal loci that are commonly reported with a CLP, although suspected loci in individual families have been hypothesised to be causal.^20^ Trisomy 21, 18 and 13 are, however, well-known associations of CLP.

Syndromic OFCs display genetic heterogeneity. A recent study^21^ of the molecular networks involved in OFCs, based on the genetic data available from a subset of 603 patients with clefting in a large cohort of patients with suspected genetic disorders in the Deciphering Developmental Disorders (DDD) study,^22^^,^^23^identified three primary molecular pathways involved in OFC: embryonic morphogenesis, protein stability and chromatin organisation. A distinction between CLP and CPO was noted in the molecular pathways, with chromatin organisation genes being implicated only in CPO. The most common single gene to be mutated was SATB2 (accounting for 2.7% of the cohort) and was specifically associated with cleft palate. The CLP group showed more genetic heterogeneity compared to the CPO group. CHD7 causing CHARGE syndrome was the most commonly reported CLP gene but was only seen in five patients. Some genes (eg CTCF, ANKRD11) were associated with both CPO and CLP, indicating more extensive functions of these genes affecting overlapping pathways implicated in both cleft subtypes.

**Fig. 1:**
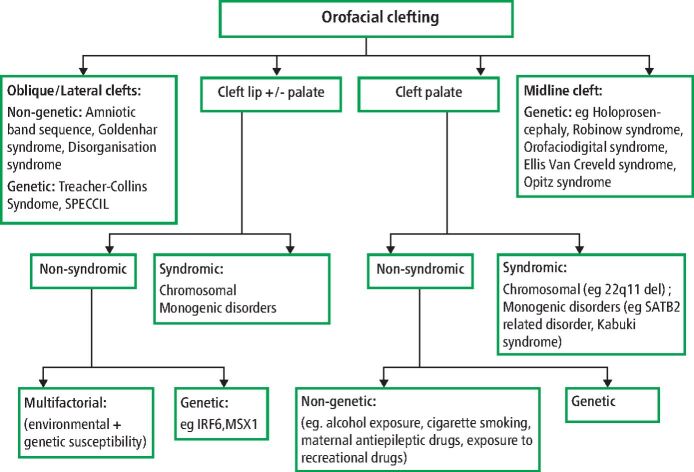
Aetiological classification of clefts

**Fig. 2:**
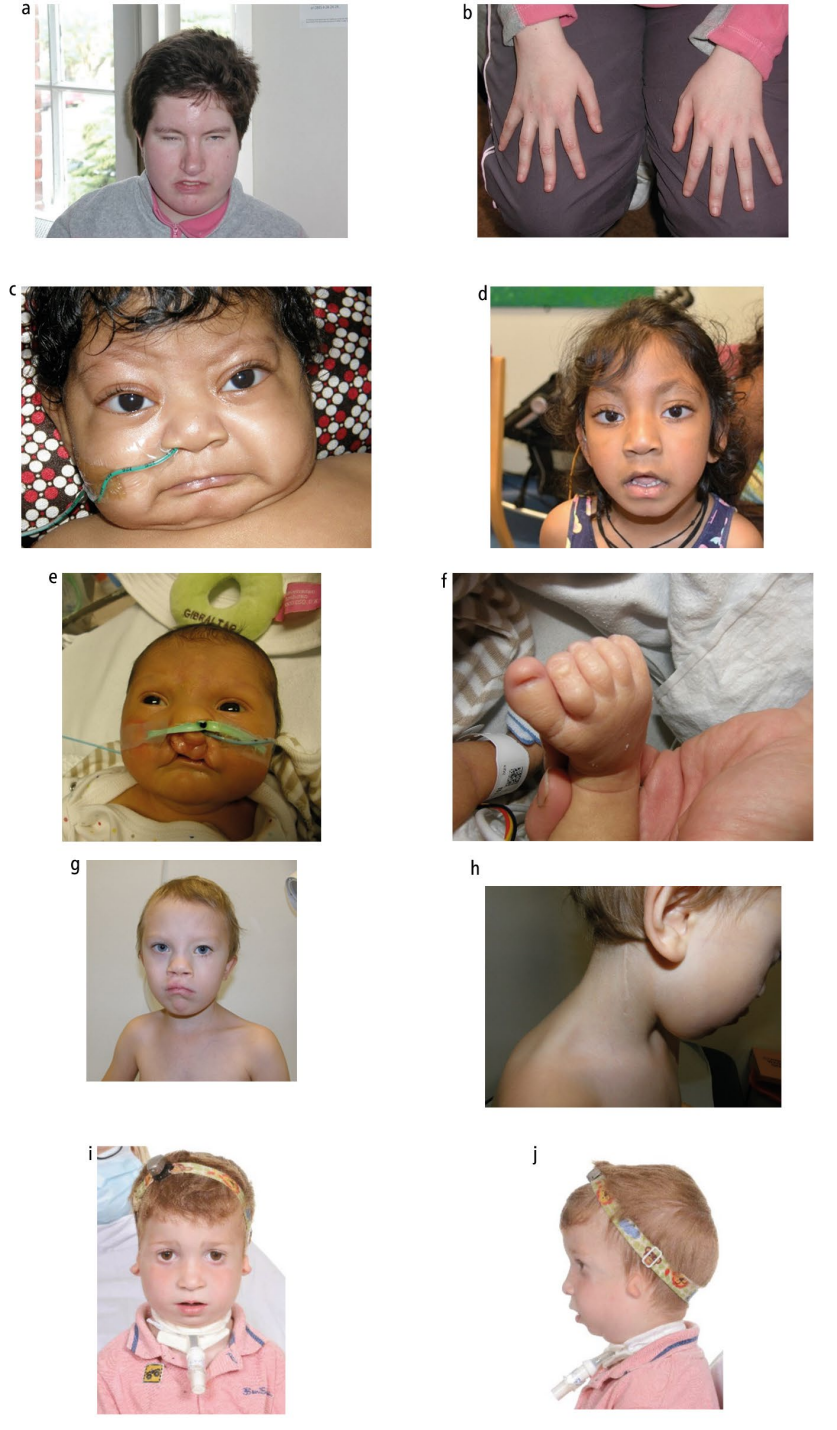
Syndromic orofacial clefts. a, b) DiGeorge syndrome: an adult showing the typical facial features of narrow eyes and the characteristic nose; long fingers also shown. c, d) Kabuki syndrome: showing characteristic facial features of interrupted eyebrows and long palpebral fissures with lateral eversion of lower eyelids with a small chin. e, f) CHARGE syndrome (coloboma, heart defect, atresia of the choanae, renal anomalies/retardation of growth and development, genitourinary anomalies and ear anomalies). g, h) Branchio-oculo-facial syndrome: showing bilateral cleft lip and palate and repaired branchial anomaly. i, j) Treacher Collins syndrome: child with micrognathia needing a tracheostomy, ear anomalies, hearing loss and flat malar region. The absence of an overt lateral cleft lip or macrostomia indicates the variability of the presentation in this disorder

Figure 1 shows the aetiological classification of clefts and Figure 2 shows examples of syndromic clefts.

## Anomalies most commonly associated with OFCs

In the DDD study,^21^ the most common associations, unsurprisingly, were under the Human Phenotype Ontology^24^ term 'abnormalities of the head and neck' and included terms referring to facial dysmorphology, such as 'hypertelorism' and 'micrognathia'. This was followed closely by 'abnormalities of the musculoskeletal system', also reported in the study by Stoll *et al.*^25^ CPO was, in particular, commonly seen in syndromes where genes affected neurodevelopment.

Dental anomalies are commonly reported with OFCs and include genes known to cause both CLP and CPO. Oligodontia is most commonly reported but other dental anomalies, if present, may inform clinical diagnosis of a genetic syndrome, for example, single central incisor indicating holoprosencephaly or macrodontia indicating KBG syndrome. Table 1 summarises the dental anomalies reported in some common cleft syndromes.^26^

**Table 1:** Dental anomalies reported in OFC syndromes

Anomaly	22q11	SATB2	Kabuki	ODD	EEC	VWS	MSX1	HPE	ANKRD11
Neonatal teeth	-	-	+	-	-	+	-	-	-
Delayed tooth eruption	+	-	-	-	-	-	-	-	-
Premature loss	-	-	-	+	-	-	-	-	-
Oligodontia	+	+	+	+	+	+	+	+	+
Abnormally shaped teeth	+	+	+	-	+	-	-	-	+
Irregular/crowded teeth	+	+	-	-	-	-	-	+	-
Small teeth	-	-	-	+	+	-	-	-	-
Malocclusion	-	+	-	-	-	-	-	-	-
Enamel abnormalities	+	-	-	+	+	-	-	-	+
Single central incisor	+	-	-	-	-	-	-	+	-
Macrodontia	-	-	-	-	-	-	-	-	+
Key: 22q11 = DiGeorge syndrome or velocardiofacial syndrome SATB2 = SATB2-associated disorder or Glass syndrome ODD = Oculo-dento-digital syndrome EEC = Ectrodactyly-ectodermal dysplasia clefting VWS = Van der Woude syndrome HPE = Holoprosencephaly ANKRD11 = Gene causing KBG syndrome									

## Genetic testing strategy

### Postnatal testing

In England, the first line of genetic testing for syndromic clefting is microarray comparative genomic hybridisation (CGH). This compares the patient's DNA to normal DNA and identifies submicroscopic deletions or duplications of chromosomes that may account for the clefting, for example, chromosome 22q11 deletion/duplication. In parts of the world where microarrays may not be easily accessible, at least a karyotype (examining the chromosomes under the microscope) and a FISH (fluorescent *in situ* hybridisation) test for 22q11 is recommended.

Further genetic testing for monogenic disorders is now available via whole genome sequencing, where a panel of genes related to clefting are analysed. Currently, there are a carefully curated 147 green genes on the panel, which is regularly updated.^27^ The PanelApp^27^ operates a traffic light system where green genes represent those with robust scientific evidence of pathogenicity (eg where there are at least three unrelated families who carry a pathogenic variant in the same gene). The amber and red genes refer to less well-established causes of clefting.

Genetic testing for non-syndromic clefting may be considered when there is a strong family history of isolated clefts. Currently, these genes are included in the 147 cleft-related genes panel.

### Prenatal testing

During a pregnancy, an orofacial cleft may be identified on an antenatal scan, usually at around 20 weeks gestation on the foetal anomaly scan. Although a cleft lip may be identified on routine ultrasound scans, a cleft palate will be missed due to the anatomical complexity. Foetal magnetic resonance imaging scans and 3D/4D scans may be offered to know the extent of the cleft, but rarely alter the management of the pregnancy.

If another additional anomaly is detected on the ultrasound scan, then genetic testing may be offered during the pregnancy as it may indicate a syndromic cleft. This is usually in the form of an amniocentesis, where foetal cells in the amniotic fluid are tested for common chromosomal anomalies and if normal, microarray comparative genomic hybridisation and foetal exome sequencing (R21) are considered based on the additional anomalies.^27^ With isolated bilateral cleft lip, there would be a low threshold for offering genetic testing.

Another indication for prenatal testing for syndromic clefting is when the pregnancy is at an increased risk of being affected due to family history. Prenatal testing is available in such a situation, only if a clear, genetically confirmed diagnosis has been established in the affected individual. In such cases, chorionic villus sampling (CVS) is offered after 11 weeks of pregnancy and foetal DNA tested for the same familial genetic variant.

Both amniocentesis and CVS have an increased risk of miscarriage (~0.2%) as these are invasive procedures. Prenatal testing is therefore recommended only in cases where it will alter the course or management of the pregnancy. Box 1 shows the prenatal and postnatal strategies for genetic testing of OFC

Box 1 Genetic testing strategy. Outlines testing available in England but practices may vary according to local accessibility to genetic testing (QF-PCR = quantitative fraction polymerase chain reaction) **Prenatal**

1. Foetal anomaly scan findings of cleft lip (CL)
2. Isolated CL:
  Unilateral - no testing recommended
  Bilateral - QF-PCR for trisomies/microarrays on amniotic fluid sample
3. CL with additional features:
  QF-PCR/microarrays for chromosomal anomalies
  Sanger sequencing/cleft panel testing/exome/whole genome sequencing for monogenic causes - on amniotic fluid sample or CVS (gross anomalies may be detected prior to the foetal anomaly scan at ~20 weeks)
4. Family history of OFCs
5. Non-syndromic OFCs - no testing recommended
6. Syndromic OFCs - testing of familial variant by Sanger sequencing if pregnancy is at risk - by CVS

**Postnatal**

1. Non-syndromic:
2. Without family history - no testing recommended
3. With family history - microarrays for chromosomal anomalies, Sanger sequencing/cleft panel testing/exome/whole genome sequencing for monogenic causes - on blood DNA
4. Syndromic:
5. Without family history - microarrays for chromosomal anomalies, Sanger sequencing/cleft panel testing/exome/whole genome sequencing for monogenic causes - on blood DNA
6. With family history - testing strategy dependent on familial genetic diagnosis - microarrays for chromosomal anomalies, Sanger sequencing for monogenic disorders - on blood DNA

### Benefits of genetic testing

There are several benefits of offering genetic testing when a syndromic cleft is suspected. Firstly, it offers an explanation for the patient's problems, if a diagnosis is made. Many parents have feelings of guilt when they have a child born with congenital anomalies. When a genetic cause is identified, it often helps relieve that sense of guilt by offering an alternate explanation. Secondly, the diagnosis allows clinicians to broadly predict the long-term prognosis for the affected individual. Thirdly, it enables the inclusion of appropriate screening measures in the long-term management of the patient, for example, a patient newly diagnosed with Kabuki syndrome should be offered a renal ultrasound, echocardiogram, ophthalmic check and long-term follow-up with a paediatrician to monitor the learning and behavioural difficulties.^28^ Additionally, a confirmed genetic diagnosis allows patients to access appropriate supportive measures, such as physiotherapy, speech and language therapy, occupational therapy and educational support, more readily. Another important benefit is the accurate prediction of recurrence risks and access to procedures that can avoid the birth of an affected child.

### Recurrence risks and reproductive options

A confirmed genetic diagnosis allows accurate prediction of recurrence risks. When a genetic diagnosis is made, it is important to establish the inheritance pattern in the family by offering parental testing for the variant identified. For those couples with an increased risk of having another affected child, prenatal testing may be offered. A termination of pregnancy may be considered for those disorders with a poor outlook.

Pre-implantation genetic testing for monogenic disorders is also available as a tool to avoid an affected pregnancy. By this procedure, following *in vitro* fertilisation, only selected embryos that do not carry the familial genetic change are implanted in the uterus. This testing is available in the NHS for couples who do not have a previous healthy child but can be accessed privately by other couples.

For genetic forms of non-syndromic clefting, the penetrance of the disease is reduced. It may therefore appear to skip a generation or more in some families. Although most genetic non-syndromic clefting is inherited in an autosomal dominant manner (that is, 50% risk of inheriting the genetic change), the actual chance of developing a cleft is much smaller. Prenatal genetic testing for non-syndromic forms is not recommended as the reduced penetrance makes it difficult to predict the outcome. Additionally, an isolated OFC does not meet the criteria for legal termination of pregnancy.

The recurrence risks for syndromic disorders depends on the inheritance pattern. For non-syndromic disorders, empiric risks may range from 2-10% based on the relationship to the index case and whether the cleft is unilateral or bilateral.^29^

### Preventative measures

Antenatal folic acid supplements have received much attention in the prevention of clefts.^25^In those with a genetic form of cleft, there is no evidence to indicate that these will alter the course of disease; monogenic causes of clefting are rarely linked to the folate metabolism pathway. In the isolated, non-genetic forms with a multifactorial aetiology, intake of normal dose folic acid (400 micrograms/day) may be protective. In fact, high dose folate supplements (5 mgs/day) are recommended with maternal anti-epileptic medication. It is preferrable to take the supplement from about six weeks pre-conception and at least through the first trimester. Avoidance of alcohol, smoking, recreational drugs and drugs with teratogenic effects is also recommended.

## Conclusion

Although current genetic testing strategy is extremely sophisticated and has substantially reduced the length of the diagnostic journey for patients with orofacial clefting, many cases still remain unsolved. This is mainly due to our current limited scientific knowledge of the function of our genome. Cleft syndromes fall under the rare diseases and ultra-rare diseases category and many of these may be caused by novel genes not as yet described to cause human disease. Another reason for failing to reach a diagnosis, despite sequencing the whole genome, is the possibility of rarer mechanisms of disruption of the gene structure or function, for example, rearrangements or methylation abnormalities,^30^ which need special analytical methods that are not inbuilt into the bioinformatics pipelines as of yet.^31^

As the healthcare system focuses increasingly on personalised and precision medicine, it will become more important to decipher the genetic causes of clefting. In the future, it could be anticipated that the individualised therapy for patients will be dependent on the genetic basis of their disease. There is already progress being made in terms of clinical trials for genetic therapies, repurposing of drugs, and small molecule therapy for some rare diseases. Data from large cohorts of OFC patients, such as that in the Cleft Collective Study (http://www.bristol.ac.uk/dental/cleft-collective/about/), should be interrogated further for genomic targets.

In the future, newborn screening is likely to include whole genome sequencing,^33^ resulting in early diagnosis of genetic causes of clefts. This will allow therapies to be instituted early and symptoms controlled, should such therapy be available. If this line of testing is extended to foetuses, it may lend itself to intrauterine interventions, such as surgical repair or gene therapy.
